# Dual effects of brain sparing opioid in newborn rats: Analgesia and hyperalgesia

**DOI:** 10.1016/j.ynpai.2018.01.001

**Published:** 2018-01-10

**Authors:** Gong Kerui, Luc Jasmin

**Affiliations:** Department of Oral and Maxillofacial Surgery, University of California San Francisco, San Francisco, CA, United States

**Keywords:** Peripheral analgesia, Neonatal, Loperamide, Blood-brain barrier, Opiate induced hyperalgesia

## Abstract

Effective pain management in neonates without the unwanted central nervous system (CNS) side effects remains an unmet need. To circumvent these central effects we tested the peripherally acting (brain sparing) opioid agonist loperamide in neonate rats. Our results show that: 1) loperamide (1 mg/kg, s.c.) does not affect the thermal withdrawal latency in the normal hind paw while producing antinociception in all pups with an inflamed hind paw. 2) A dose of loperamide 5 times higher resulted in only 6.9 ng/mL of loperamide in the cerebrospinal fluid (CSF), confirming that loperamide minimally crosses the blood–brain barrier (BBB). 3) Unexpectedly, sustained administration of loperamide for 5 days resulted in a hyperalgesic behavior, as well as increased excitability (sensitization) of dorsal root ganglia (DRGs) and spinal nociceptive neurons. This indicates that opioid induced hyperalgesia (OIH) can be induced through the peripheral nervous system. Unless prevented, OIH could in itself be a limiting factor in the use of brain sparing opioids in the neonate.

## Highlights

• The peripherally acting opioid loperamide produces sustained antinociception in the newborn rat.
• Loperamide minimally crosses the blood brain barrier in the newborn rat.
• Daily systemic administration of loperamide produces opioid induced hyperalgesia in the newborn rat.

## Introduction

Unrelieved pain in the term and preterm neonate initiates maladaptive plasticity that can persist later in life (Schwaller and Fitzgerald, 2014, Walker et al., 2016). Opioids can prevent this plasticity while providing analgesia. There are concerns, however, that opioids have unwanted effects on the immature brain (Attarian et al., 2014, Beltran-Campos et al., 2015, de Graaf et al., 2011, Ferguson et al., 2012, Rozisky et al., 2011). For instance preemies who received opiates in the neonatal intensive care unit (NICU), can develop a smaller head-circumference, lower body weight, short-term memory impairments, and difficulty socializing (Attarian et al., 2014, Ferguson et al., 2012). In animal models, administrating opioids during the post-natal period leads to altered mu-opioid receptors (MORs) expression in the forebrain (Handelmann and Quirion, 1983), and increased pain behavior later in life (Rozisky et al., 2011). Given that opioids are effective analgesics for acute pain, a possible strategy is to use brain sparing (peripherally acting) opioids in the newborn. To explore this approach we chose the brain sparing MOR agonist loperamide (Guan et al., 2008, Kumar et al., 2012, Nozaki-Taguchi and Yaksh, 1999). Loperamide produces analgesia in adult models of inflammatory (Shannon and Lutz, 2002), cancer, and neuropathic pain (Chung et al., 2012, Guan et al., 2008) by acting on the peripheral opioid receptors (DeHaven-Hudkins et al., 1999, Guan et al., 2008). Accordingly, MORs in the periphery are critically involved in the analgesic effects of opioids (Taddese et al., 1995, Wang et al., 2010). Since there is a greater expression of MORs in primary sensory neurons during the first 2 post-natal weeks (Beland and Fitzgerald, 2001, Nandi et al., 2004), we postulated that newborns would be ideal candidates for loperamide induced antinociception. We tested loperamide in newborn rats, which are developmentally similar to premature humans (Romijn et al., 1991, Sengupta, 2013). We first assessed the effects of loperamide on the nociceptive withdrawal threshold in normal newborns, and then in newborns with an inflamed hind paw after a local carrageenan injection (Fehrenbacher et al., 2012). We then determined if loperamide crosses the blood–brain barrier (BBB) of the neonate rat. Finally, given that brain penetrant opioids can produce pro-nociceptive effects (Roeckel et al., 2017), we tested the effect of daily loperamide on the nociceptive threshold, the peripheral neuronal activity using patch clamp recordings, and the CNS activity using Fos immunochemistry.

## Materials and methods

### Experimental animals

Male and female Sprague-Dawley rats (Charles River Lab, USA), post-natal day 3 (P3) at the start of the experiment, were studied. Pups were kept with their littermates and mother in a dedicated room with alternating 12 h of light-dark cycle. Food and water were available *ad libitum*. For each experimental group, 8–10 pups were used. No adverse effects of loperamide were observed during the experiment.

### Ethics

Procedures for the maintenance and use of the experimental animals conformed to the regulations of UCSF Committees on Animal Research and were carried out in accordance with the guidelines of the NIH regulations on animal use and care (Publication 85–23, Revised 1996). The UCSF Institutional Animal Care and Use Committee approved the protocols for this study.

### Experimental protocols

Loperamide and chemicals were purchased from Sigma-Aldrich unless noted otherwise.

For acute experiments, a single dose (1 mg/kg, s.c.) of loperamide 1 mg/mL or equal volume of vehicle (sterile 5% DMSO) was administered 30 min before carrageenan (1% in 0.9% saline, 20 μl, intradermal with a 30 ga needle) in the left hind paw. This preemptive analgesia mimics protocols promoting early interventions (drugs or others) in the NICU to prevent the long-term effects of untreated pain (Cignacco et al., 2009, Cruz et al., 2016, Laprairie et al., 2008).

**Fig. 1:**
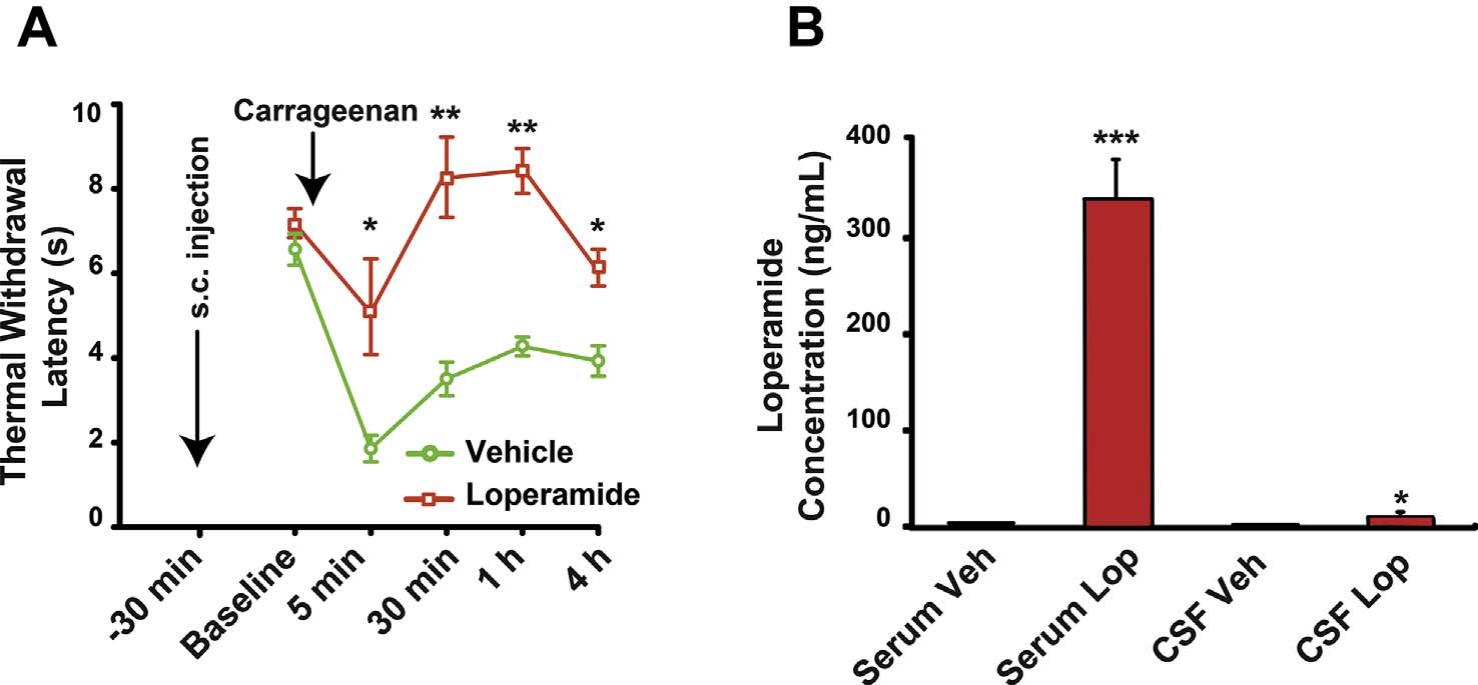
Effect of a single dose of loperamide on thermal withdrawal latency, and systemic vs. central distribution. (A) Loperamide (1 mg/kg) or its vehicle were injected s.c. and 30 min later carrageenan (1% in 0.9% saline, 20 μl, intradermal) was injected in the left hind paw. The antinociceptive effect of loperamide was then monitored for the following 4 h (n = 10 for both groups) using the Hargreaves plantar test. P values are obtained after comparing Vehicle vs. Loperamide groups at each time point. (B) Concentrations of loperamide in the serum and CSF. Mass spectrometry showed that loperamide poorly penetrates the blood-brain barrier in neonates (P3). Six hours following 5 mg/kg, s.c., the concentration of loperamide in serum was 334 ng/mL, while in the CSF it was 6.9 ng/mL. P values are obtained by comparing the concentration of each treatment group with the 3 others. * P < .05, ** P < .01, *** P < .001.

Prior to the injection of carrageenan, but not prior to loperamide (Fig. 1A), rats were tested for the baseline thermal withdrawal latency (Hargreaves plantar test). In preliminary experiments we observed that loperamide 1 mg/kg did not increase the withdrawal latency in the Hargreaves test. We also found that decreasing the number of heat exposures in neonates minimizes the risk of stimulus induced paw sensitization. Rats were then retested at 5 min, 30 min, 1 h and 4 h after the carrageenan injection.

For chronic experiments, loperamide was administered once daily (1 mg/kg, s.c.) starting at P3 lasting until P7 (total of 5 days). Hind paw withdrawal latency to the heat stimulus was evaluated everyday starting on the first day prior to the initial dose of loperamide and then daily 6 h after each injection. This delay of 6 h, between the loperamide injection and the Hargreaves test, ensured that the nociceptive threshold was measured when the plasma levels of loperamide were high (He et al., 2000, Heel et al., 1978, Killinger et al., 1979, Miyazaki et al., 1979, Streel et al., 2005). Testing animals immediately prior to the daily injection of loperamide might have also showed hyperalgesia, whereas it could have been part of an early opioid withdrawal instead (Lee et al., 2011).

On each day, after pups were administered loperamide or tested, they were immediately returned to the dam. Precautions were taken to ensure that none of these newborns were rejected by their mother. During all manipulations and testing procedures, care was taken to maintain body temperature constant.

Control animals received the same volume of vehicle (sterile 5% DMSO) on the same schedule. After the last dose of loperamide or vehicle, pups (P7) were randomly injected with carrageenan or saline (20 μl, intradermal) in the left hind paw. Their lumbar spinal cord was collected and processed for Fos immunocytochemistry 3 h later.

### Heat sensitivity (Hargreaves plantar test)

An investigator blind to the treatment groups performed the behavioral studies. Heat pain latency was measured using the Hargreaves plantar test device (Harvard apparatus, USA) (Cheah et al., 2017). Rats were placed into the test area 60 min prior to testing. The glass plate on which they were free to move was preheated to 30 °C to keep them comfortable. The withdrawal latency from a heat stimulus was measured 3 times for each hind paw, with a 5-min interval between individual measures. The mean value in seconds was used as the thermal nociceptive threshold. Although never reached, a cutoff of 20 s was used to prevent skin damage.

### Biological fluid samples

To assess for possible penetration of loperamide in the CNS, we determined the concentration of loperamide in the CSF in P3 rats (n = 8) using mass spectroscopy (Rubelt et al., 2012). Serum levels were also determined by the same method. Based on a plasma half-life of 9–13 h (Doser et al., 1995, Killinger et al., 1979, Yu et al., 2004), a time to peak plasma concentration of 2.5 to 6 h (He et al., 2000, Heel et al., 1978, Killinger et al., 1979, Miyazaki et al., 1979, Streel et al., 2005), and a duration of action of up to 3 days (Heel et al., 1978), CSF and blood samples were acquired 6 h after a high dose of loperamide (5 mg/kg, s.c.).

CSF was obtained by puncture of the dura overlying the cisterna magna using an operating microscope and a pulled glass capillary pipette while the animals were under hypothermic anesthesia (Liu and Duff, 2008). Care was taken to make sure that the CSF was not contaminated by blood. Collection of blood was done by cardiac puncture into a 1.5 mL tube containing EGTA. The blood was spun down at 1500 g for 10 min in a refrigerated centrifuge. The supernatant (serum) was collected into a clean tube. CSF and serum samples were kept at −20 °C prior to analysis.

Serum and CSF loperamide levels were determined by liquid chromatography-tandem mass spectrometry (LC-MS/MS) using Agilent LC 1260-AB Sciex 5500 (binary pump, Agilent, USA). Each analyte was ionized using electrospray ionization in the negative mode and monitored by multiple reactions. The serum and CSF were prepared for LC-MS/MS analysis by solid phase extraction using Waters Oasis HLB cartridge (10 mg, 1 mL). Each cartridge was washed with 5 column volumes of methanol prior to activation with water for loading of serum or CSF. The column was washed with 1 mL 5% methanol before each analyte was eluted with 1 mL of methanol. The eluates were evaporated under a stream of nitrogen gas after which they were reconstituted in 10% methanol for column injection. A 5 μl aliquot of the extract was used for each replicate injection of the sample. Chromatographic separation of the analytes was achieved by gradient elution using MeOH/H_2_O (97/3, v/v) + 10 mM ammonium acetate + 0.1% acetic acid as solvent A, and MeOH/H_2_O (10/90, v/v) + 5 mM ammonium acetate + 0.1% formic acid as solvent B. The elution gradient employed was 0–0.5 min = 30% B; 0.5–1 min = 75% B; 1–4 min = 100% B; 4–5.5 min = 100% B; and 5.5–6 min = 30% B. The analytes had a quantitation limit of 0.1 ng/mL (part per billion). Data analysis was conducted using AB Sciex Analyst 1.6 and AB Sciex MultiQuant 2.1 software packages.

### Patch clamp recordings

The method for intact DRG recordings has recently been described (Gong et al., 2016b). This method preserves the neuroglial interactions as well as the afferent and efferent axons to obtain data closer to *in vivo* conditions. On P7 (on the 5th day of daily loperamide administration) neonatal rats were euthanized and the spines were quickly removed. The spines were then placed into ice-cold carbogenized artificial CSF (aCSF). The aCSF contained: 124 mM NaCl, 2.5 mM KCl, 1.2 mM NaH_2_PO_4_, 1.0 mM MgCl_2_, 2.0 mM CaCl_2_, 25 mM NaHCO_3_, and 10 mM glucose. Laminectomies were performed and the spinal cords were removed. Following this, the DRGs were collected under the dissection microscope (Wild, Germany). Each DRG was transferred to a recording chamber after the surrounding connective tissue was removed. There, it was perfused with aCSF at a rate of 2–3 mL/min. A small area of the collagen layer on the surface of each DRG was digested to expose neurons to the recording pipette. For this purpose we used the enzyme mix “Liberase” (Roche, USA). A fine mesh anchor (SHD-22L, Harvard, USA) was used to anchor down the DRGs during recordings.

DRG neurons were visualized with a 40X water-immersion objective using a microscope (FN-600; Nikon, Japan) equipped with infrared differential interference contrast optics. The image was captured with an infrared-sensitive CCD (IR-1000, Dage MTI, USA) and displayed on a black and white video monitor. Currents were recorded with an Axon 200B amplifier (Molecular Devices, USA) connected to a Digidata interface (Digidata 1322A, Molecular Devices, USA) and low-pass filtered at 5 kHz, sampled at 1 kHz, digitized, and stored using pCLAMP 10.2 (Molecular Devices, USA). Patch pipettes were pulled from borosilicate glass capillary tubing (BF150-86-10, Sutter, USA) with a P97 puller (Sutter, USA). The resistance of the pipette was 4–5 MΩ when filled with recording solution which contained: 140 mM KCl, 2 mM MgCl_2_, 10 mM HEPES, 2 mM Mg-ATP, 0.5 mM Na_2_GTP, pH 7.4. Osmolarity was adjusted to 290–300 mOsm. After a gigaseal was established on a neuron, the membrane was broken and the cell was selected for further study if it had a resting membrane potential of less than −50 mV. The access resistance was 10–20 MΩ and continuously monitored. Data were discarded if the access resistance changed more than by 15% during an experiment. Small diameter neurons, i.e. dark neurons, were exclusively selected for patch clamp recordings (Gong et al., 2016a, Gong and Jasmin, 2017, Lawson, 1979). The size of the neurons was determined by measuring the diameter on the screen.

### Tissue preparation and immunostaining protocol

The DAB method was used to label Fos positive cells in the lumbar spinal dorsal horn. Three hours after an intradermal injection of 20 μl 1% carrageenan or vehicle, rats were perfused intracardially with physiologic saline, followed by 10% formalin, pH 7.4. The L3-5 spinal cord segments were processed for immunostaining. The samples were post-fixed for 2 h and then placed in a 30% buffered sucrose solution overnight. Ten micron transverse sections were cut with a cryostat. Fos immunostaining was then performed. Briefly, after blocking by 10% normal goat serum in phosphate buffered saline (PBS) with 0.3% Triton X-100 (PBS-TX) for 1 h at room temperature, the sections were incubated with the anti-Fos primary antibody (1:20,000, rabbit, a gift from Prof. Dennis Slamon, UCLA) for 24 h at 4 °C. The sections were then rinsed and incubated with biotinylated anti-rabbit secondary antibody (1:500, Sigma, USA) for 1 h at room temperature. The sections were rinsed again and incubated with Extravidin (1:100, Sigma, USA) for 1.5 h. Then a DAB kit (Sigma, USA) was used for final staining of Fos. Sections were put under the dissection microscope for visual determination of the reaction time. We used ultra pure water to end the reaction. The sections were dehydrated and covered for further analysis.

### Cell counting

Counts of Fos-labeled cells were made on 6 randomly selected lumbar spine sections for each rat. The investigator responsible for plotting and counting the labeled cells was blind to the drug treatment of individual animal. The superficial dorsal horn of the spinal cord was identified using dark-field illumination (Molander et al., 1984, Steiner and Turner, 1972). A nucleus was counted as Fos positive if it was entirely filled with black reaction product. Based on nuclear size, cell shape, and extensive experience of our laboratory with this technique, we determined that the Fos positive cells counted were neurons.

### Statistical analysis

All results are presented as the mean ± SEM. For the analysis of thermal threshold, repeated-measures one-way ANOVA followed by Bonferroni post hoc or Student’s *t*-test were used. For patch clamp recordings, the Student's *t*-test was used. For the Fos labeled cell counts, statistical comparisons were performed using the Student’s *t* test (unpaired, two tailed) to compare the means between groups. Differences between means were considered statistically significant at P < .05.

## Results

### Dose of loperamide and body weights

Based on preliminary experiments and data from Guan and colleagues (Guan et al., 2008), we used 1 mg/kg, s.c. of loperamide for behavioral experiments. The average body weight of pups was 9 ± 0.5 g at P3, 11.1 ± 0.6 g at P4, 14.9 ± 0.4 g at P5, 17.2 ± 0.6 g at P6, and 19.8 ± 0.5 g at P7. Administration of loperamide for 5 consecutive days did not affect the body weight when comparing with the standard growth curve (Yuan et al., 2000).

### Antinociceptive effect of loperamide

A single injection of loperamide (1 mg/kg s.c.) did not prolong the paw withdrawal latency compared to vehicle in the Hargreaves test (7.1 ± 0.5 s vs. 6.5 ± 0.4 s; p > .05; n = 10 in each group) (Fig. 1A). This is consistent with the effect of 1 mg/kg of morphine in adult rats submitted to the Hargreaves test (Morgan et al., 2006). Subsequent intraplantar injection of carrageenan, to produce a local inflammation, exposed the antinociceptive effect of loperamide. Rats were injected with carrageenan 1% (20 μl) in the left hind paw and were tested 5 min, 30 min, 1 h, and 4 h later (Fig. 1A). At 5 min, vehicle treated rats had a marked decreased withdrawal latency from 6.5 ± 0.4 s to 1.9 ± 0.3 s (p < .05). Loperamide produced significant antinociception at all time points, with some remaining nociception at 5 min when comparing pre- (7.1 ± 0.5 s) vs. post-carrageenan (5.1 ± 1.2 s) withdrawal latencies (Fig. 1A).

### Concentration of loperamide in the serum and CSF

To determine if loperamide penetrates the BBB after systemic administration, we injected a single dose of 5 mg/kg, s.c. and measured the concentration of loperamide in the serum and the CSF 6 h later (n = 8) using mass spectrometry. The serum concentration of loperamide was 334.7 ng/mL while in the CSF concentration was only 6.9 ng/mL of loperamide, which is about 50 times less (Fig. 1B).

### Loperamide induced hyperalgesia and hyperexcitability of sensory neurons

**Fig. 2:**
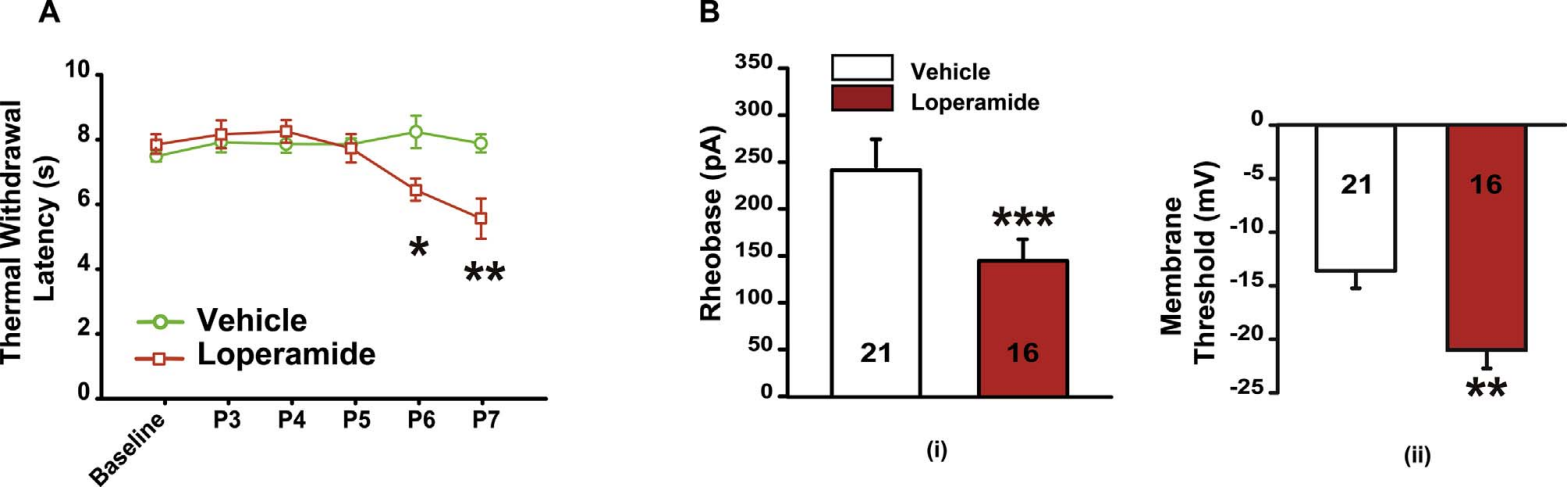
Effect of daily administration of loperamide (1 mg/kg, s.c.) for 5 days (P3 to P7) on nociceptive paw withdrawal latency and DRG neuron membrane conductance. (A) Behavioral hyperalgesia to a heat stimulus appeared on the 4th day (P6) of loperamide administration (P values are obtained by comparing Vehicle vs. Loperamide groups at each time point). (B) Patch clamp recordings of small diameter DRG neurons were done on the 5th day (P7) of loperamide administration. Neurons in the loperamide group showed lower rheobase (i) and membrane threshold (ii) compared with the vehicle group. Numerals in each column stand for the number of neurons recorded. n = 10 rats for both groups; * P < .05, ** P < .01, *** P < .001.

To further investigate whether loperamide could induce opioid induced hyperalgesia (OIH) in the neonates, just as morphine does (Zhang and Sweitzer, 2008, Zissen et al., 2006, Zissen et al., 2007), we administered loperamide daily (1mg/kg, s.c.) from P3 to P7 and performed daily Hargreaves plantar tests. Compared to rats receiving vehicle (n = 10), those receiving loperamide (n = 10) did not show any significant change in the withdrawal latency from the nociceptive stimulus during the first 3 days (P3 to P5). However, starting at P6, the loperamide group exhibited a significantly decreased latency with an average latency of 5.8 ± 0.3 s compared to the vehicle group with an average latency of 7.8 ± 0.4 s (Fig. 2A, p < .05). This difference between the two groups was accentuated on P7 with an average withdrawal latency of 5.1 ± 0.6 s for the loperamide group vs. 7.6 ± 0.3 s (p < .01) for the vehicle group.

The lumbar DRGs from the above rats were then collected on P7. Patch clamp recordings on small diameter neurons (<30 µm) were conducted on whole DRGs as we previously reported (Gong et al., 2014, Gong et al., 2016a, Gong et al., 2016b, Gong and Jasmin, 2017). DRG neurons from the loperamide group (treated for 5 consecutive days) demonstrated an average current threshold of 145.1 ± 15.1 pA, which was significantly lower than that of the vehicle treated group (240.2 ± 39.7 pA, p < .001, Fig. 2Bi). Also, in the loperamide group the membrane threshold was reduced to –22.1 ± 3.9 mV from −14.1 ± 2.7 mV (vehicle treated group, p < .01, Fig. 2Bii).

**Fig. 3:**
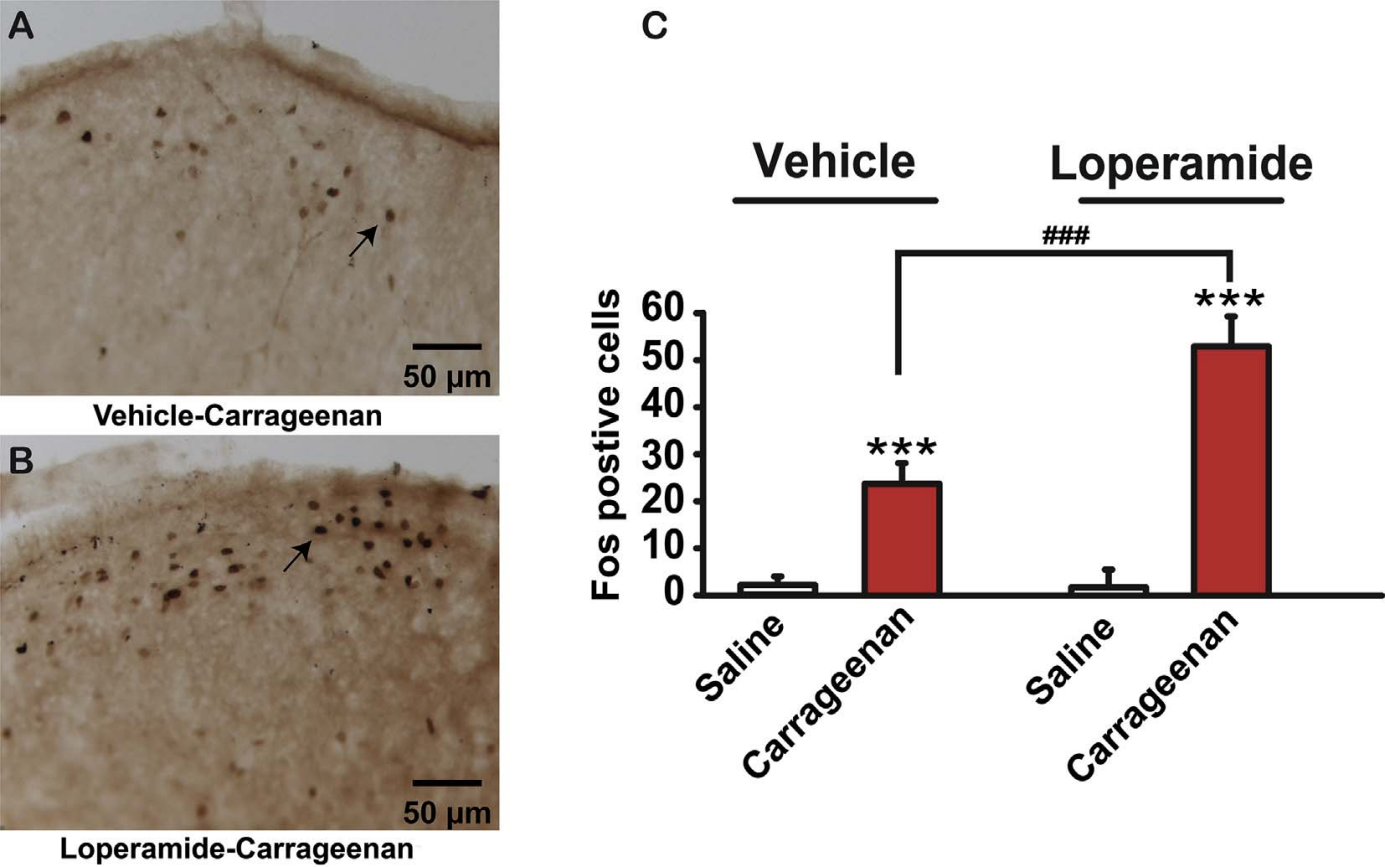
Fos expression in the lumbar spinal cord following daily administration of loperamide (1 mg/kg, s.c.) for 5 days. Photomicrographs of representative sections of the superficial dorsal horn from vehicle-carrageenan (A), and loperamide-carrageenan (B) treated pups show greater number of Fos positive neurons for loperamide treated pups. Arrows in A and B point to Fos positive neurons. In C, the histogram shows the average number of Fos positive cells for individual treatment groups. Within each treatment group (Vehicle or Loperamide) the number of Fos cells after a saline vs. carrageenan stimulus is compared (white bars vs. red bars) and the p values are showed with stars (*). A second level of comparison is made between the Vehicle and Loperamide, and the p values are showed with hash tags (#). *** P < .001, ### P < .001. Data are from 8 animals per group. (For interpretation of the references to colour in this figure legend, the reader is referred to the web version of this article.)

Finally, we sought to determine if loperamide induced hyperexcitability of primary sensory neurons would result in a greater activation of spinal superficial dorsal horn neurons, where primary nociceptive afferents terminate. Rats were treated with loperamide or vehicle for 5 days (P3 to P7, 1 mg/kg, s.c., n = 10). Six hours after the last dose of loperamide or vehicle, carrageenan 1% (20 μl) or its vehicle (saline 0.9%) was injected in the left hind paw, and rats were euthanized 3 h later. We did not do a heat latency testing prior to euthanasia to avoid a second stimulus, which would have been a confounding variable. The lumbar spinal cords were collected and processed for Fos immunocytochemistry. Fos positive cells were counted in the four different groups. The increased in the number of immunopositive cells was localized mainly in the superficial dorsal horn (layer I/II of Rexed). There, in vehicle-carrageenan treated rats Fos neuronal count was 25.1 ± 6.1 per section (Fig. 3A). However, in loperamide-carrageenan treated rats Fos was seen in twice as many neurons: 52.4 ± 7.5 per sections (Fig. 3B and C, p < .001). Lastly, in both vehicle and loperamide treated rats, when saline instead of carrageenan was injected in the left hind paw much fewer Fos expressing cells could be detected: 2.3 ± 1.1 and 2.4 ± 3.1 cells per section respectively (Fig. 3C).

## Discussion

Our results show that a peripherally acting opioid is antinociceptive in the newborn rat, and induces OIH within a few days with continued administration. We suggest that these effects of loperamide are enabled by an high expression of MORs in primary sensory neurons during the first 2 post-natal weeks (Beland and Fitzgerald, 2001, Nandi et al., 2004).

### Loperamide and the blood-brain barrier

We chose loperamide because it is a MOR agonist (DeHaven-Hudkins et al., 1999, Guan et al., 2008, Kumar et al., 2012, Nozaki-Taguchi and Yaksh, 1999) which does not cross the BBB (Schinkel et al., 1996). It is also antinociceptive in models of inflammatory, cancer, and neuropathic pain (Chung et al., 2012, Guan et al., 2008, Menendez et al., 2005, Shannon and Lutz, 2002). Clinically, loperamide is used mainly to treat traveler’s diarrhea and has been listed as one the fundamental drugs by the World Health Organization. Only a few reports suggest a possible analgesic effect in humans, for instance when it is applied topically (Nozaki-Taguchi et al., 2008). Limited data is found on its use during neonatal period and mainly pertains to the treatment of short bowel syndrome in the NICU (Amin et al., 2013).

In rodents and human neonates, a therapeutic dose of loperamide should produce antinociception essentially through the periphery given that the BBB is formed and functional (Daneman et al., 2010, Mollgard and Saunders, 1986, Saunders et al., 2012). In agreement, we measured only 6.9 ng/mL (parts per billion) of loperamide in the CSF of P3 rats after a high systemic dose. While statistically significant, such a low CSF concentration is unlikely to be antinociceptive since, in adult rats, at least 30 μg of intrathecal loperamide is needed to produce significant antinociception (Ray and Yaksh, 2008). Given that the total volume of CSF in an adult rat is approximately 275 μl (Chiu et al., 2012), a dose of 30 μg of loperamide should result in a CSF concentration of about 109 ng/mL, which is 15 times greater than the 6.9 ng/mL observed here. Because the CSF dosage was obtained after a dose 5 times greater than that needed to produce a robust antinociceptive effect, we suggest that the systemic dose of loperamide of 1 mg/kg in our protocol was too low to alter the pain behavior through a direct effect on the CNS.

### Opioid induced hyperalgesia (OIH)

Within days of receiving loperamide, neonates exhibited a decreased nociceptive latency, which is characteristic of OIH. This was an unexpected result and to our knowledge the first demonstration of OIH following the administration of a peripherally acting opioid. Peripherally mediated OIH, however, was recently reported in adult mice, after the combined administration of morphine and a peripherally acting opioid antagonist blocked the appearance of hyperalgesia from daily morphine (Corder et al., 2017). By using conditional knockout mice for MOR in TRPV1 neurons, the authors also concluded that DRGs nociceptive neurons are critical in the appearance of morphine associated hyperalgesia and tolerance (Corder et al., 2017). This is consistent with our results showing that peripheral sites (DRGs) are involved in OIH.

Tolerance to loperamide, in turn, was previously reported for adult rats (He et al., 2013), which is significant since it shares some, but not all, cellular mechanisms with OIH (Fang et al., 2017, Ferrini et al., 2013, Jin et al., 2017, Song et al., 2015). Also, OIH has been previously reported in neonates after repeated administration of the brain penetrant opioid morphine (Zhang and Sweitzer, 2008, Zissen et al., 2006, Zissen et al., 2007).

Repeat administration of opioids profoundly affects peripheral neuronal physiology in adult rats (Gong et al., 2016a, Gong and Jasmin, 2017). Patch clamp recordings in newborns showed similar hyperexcitability in DRG neurons, which is in agreement with our behavioral data. After 5 days of loperamide treatment, small diameter DRG neurons from P7 rats displayed increased excitability and decreased threshold. These results confirm previous studies showing that primary sensory neurons are involved in OIH (Gong et al., 2016a).

In loperamide treated rats, the increased Fos expression in the superficial dorsal horn suggests that the OIH associated increased excitability of primary sensory neurons leads to central sensitization. In fact, OIH is generally seen as a form of central sensitization (Lee et al., 2011), involving glutamate, dynorphins, descending facilitation, and greater response to nociceptive neurotransmitters (Lee et al., 2011). Recent studies suggest, however, that the peripheral nervous system is also involved. Notably, Corder and colleagues (Corder et al., 2017) recently found that opioid induced long-term potentiation (LTP) at the first synapse in the spinal cord was dependent on pre-synaptic MOR expressing nociceptive neurons. Opioid induced sensitization of peripheral nerve endings (Araldi et al., 2017) and DRG neurons (Gong et al., 2016a, Gong and Jasmin, 2017) would result from transcriptional changes and post-translational changes such as phosphorylation mediated relocalization and upregulation of receptors and ion channels (Araldi et al., 2017, Gong et al., 2016a, Gong and Jasmin, 2017). Ion channels would open more frequently and for longer times, causing increased nociceptive afferent activity. The sensitization of primary sensory neurons would result in an increase in synaptic transmission at the spinal level and ensuing plasticity. An opioid induced increase in activity of nociceptive afferents term and preterm neonate’s nervous system might shape sensory responses for life (Koch and Fitzgerald, 2013). Features that facilitate this plasticity in the newborn include: greater amounts of Ca^++^ permeable GluN2B containing NMDA receptors (NMDARs) (Monyer et al., 1994, Yashiro and Philpot, 2008), a widespread distribution of NMDARs throughout the spinal dorsal horn (Gonzalez et al., 1993, Hori and Kanda, 1994), and increased responsiveness to glutamate (Tahayori and Koceja, 2012).

All these data indicate that despite their non-brain penetrant advantage, peripherally acting opioids might still have an impact on the CNS. Since MOR is involved in the initiation but not the maintenance of OIH (Araldi et al., 2017), preventing or reversing the post-translational changes might allow maintaining the analgesic effects of opioids without OIH. This hypothesis, however, remains to be tested.

## Conclusion

We chose a peripherally acting opioid, both because neonates get significant antinociception and because the BBB can be impermeable to these drugs. Serendipitously we observed that within days loperamide treated rats developed OIH and central sensitization. These findings support the use of brain sparing opioids in the newborn. Strategies to avoid the hyperalgesic effect of peripheral opioids will need to be developed to transition to clinical trials.

## Disclosures

The authors have no conflicts of interest to declare.

## Funding

This work was supported by the Painless Research Foundation.
